# Checklist-based approach to measure milker behavior before and after training

**DOI:** 10.3168/jdsc.2023-0454

**Published:** 2023-11-17

**Authors:** Wolfgang Heuwieser, Rachel Moody, Michael Zurakowski, Paul D. Virkler

**Affiliations:** 1 Department of Population Medicine and Diagnostic Sciences, Cornell University, Ithaca, NY 14853; 2 Clinic for Animal Reproduction, Faculty of Veterinary Medicine, Freie Universität Berlin, 14163 Berlin, Germany

## Abstract

So far, evaluation of training initiatives for dairy farm employees has been limited to the reaction and learning level. The effect of training on dairy farm employees' behavior has not been studied yet. The objective of this study was to determine the effectiveness of online training to change employee behavior toward best-practice routines. An interactive training course related to udder health was developed in 5 modules in Spanish and English: (1) collecting an aseptic milk sample, (2) administering intramammary treatment, (3) deciding on salable milk, (4) treating a cow at dry-off with antibiotics, and (5) administering teat sealants. Participants navigated the modules at their own pace or watched a narrated video. Employees were assessed performing 2 procedures related to dry cow treatment (i.e., treating a cow at dry-off with antibiotics, administering internal teat sealant) by using an objective structured clinical examination. If possible, all employees were scored performing the procedure on 2 cows before and within 2 to 3 wk after the training was applied.

## Graphical Abstract

**Figure:**
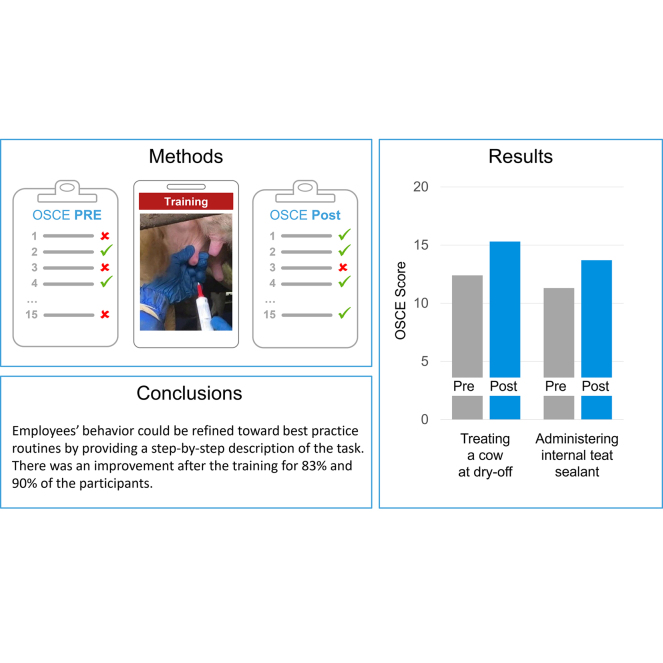

The majority of participants (72%) watched a narrated video, and 28% decided to browse the modules at their own discretion. Twenty-nine and nineteen employees were scored pre- and post-training for treating a cow at dry-off and for administering internal teat sealants, respectively. There was an improvement in the average objective structured clinical examination (**OSCE**) score (maximum of 20 points) for both modules from 12.4 (interquartile range; **IQR**: 2.5) and 11.3 (±0.95) pre-training to 15.3 (IQR: 1.0) and 13.7 (±0.93) post-training. The mean differences were 3.0 ± 0.49 and 2.4 ± 0.51. After the training, 83% and 90% of the participants had improved.

Producing high-quality milk and maintaining good udder health at the herd level is a constant challenge on many dairy farms (McMullen et al., 2021). Insufficiently trained employees lead to poor or inconsistent process quality concerning milking routines, solving equipment issues, and implementing proper udder health procedures. It is well known that there is a relationship between employee development and employee performance in a variety of business types (Lee and Bruvold, 2003; Hameed and Waheed, 2011). On dairy farms, management of employees such as goal setting, training, providing tools and performance feedback, having clear lines of supervision, and developing a team contribute to dairy employee satisfaction, recruitment, and retention success (Schuenemann, 2017; Durst et al., 2018).

The importance of training dairy employees has been documented (e.g., Barkema et al., 2015; Moore et al., 2020). Regular training of employees is considered essential to reduce procedural drift (Biagiotti, 2016; Bauer, 2023). Bilingual training has been considered as a key issue in the development of employee productivity (Gutierrez-Solano et al., 2011). Also, it has been demonstrated that training improved the knowledge of dairy personnel on calving management practices (Schuenemann et al., 2013) and was associated with improvement of SCC for 10 mo following the training course (Poirier et al., 2011).

In previous studies we have shown that dairy employees rated short online training modules focused on udder and calf health endorsing standard operating procedures (**SOP**) as positive and relevant and showed high levels of engagement. Furthermore, online training was effective to change the feeling of confidence and accuracy of work processes (Hesse et al., 2019; Alanis et al., 2022; Neukirchner et al., 2022). These findings correspond to level 1 (i.e., reaction level) and 2 (i.e., learning level) of the Kirkpatrick model (Kirkpatrick, 1996). This evaluation model comprises 4 levels (i.e., reaction, learning, behavior, and impact). So far, evaluation of training initiatives for dairy farm employees has been limited to level 1 and 2 (Hesse et al., 2019; Garzon et al., 2023).

The effectiveness of OSCE for assessment of learners' knowledge, skills, and behaviors has been demonstrated in human and veterinary medicine (Casey et al., 2009; Hall et al., 2023). In both disciplines, OSCE are accepted for clinical exams (Newble, 2004; Schlesinger et al., 2021). These tools allow objective scoring of candidates (e.g., students or employees) by observing them perform a task while evaluating all critical steps in a systematic way using a grading tool such as a checklist (Hall et al., 2023). An OSCE is best suited to testing clinical, technical, and practical skills and can be used for a broad range, often with a high degree of fidelity (Newble, 2004). The examiner is equipped with a checklist constructed of items or critical steps that the candidate should perform in a specific manner (Davis et al., 2006). The correct implementation of a given item can be rated with 0, 1, or 2 points according to importance and complexity.

Despite the demonstrated effectiveness to evaluate skills and behavior with an OSCE systematically and objectively (Zayyan, 2011), such tools have not been used to measure dairy farm employees' behavior before and after training. We hypothesized that a training effect on behavior, related to implementing udder health procedures, can be observed by comparing before and after training assessments. Specific objectives of this study were (1) to employ a method to objectively score employees by watching them perform 2 procedures related to udder health and antibiotic drug use (i.e., treating a cow at dry-off, administering internal teat sealant) while evaluating critical steps, in a systematic way, by using an OSCE checklist and (2) to compare the scores before and after training of those tasks by means of an interactive online course.

An interactive online training course on udder health and prudent antibiotic drug use was developed with a cloud-based authoring tool (Visme, Rockville, MD). The course consisted of 5 modules in Spanish and English: (1) collecting an aseptic milk sample, (2) administering intramammary treatment, (3) deciding on salable milk, (4) treating a cow at dry-off, and (5) administering teat sealants.

Each module consisted of 3 sections describing the materials (What do I need?), critical steps (How do I do it?), and background information (Why is it important?). The last section is particularly important as learners need to understand why certain steps need to be executed in a certain way to truly understand the importance of their work (Roman-Muniz et al., 2007).

The first section listed the materials required to implement the task and showed an appropriate picture. For section 2, a given task was dissected into 12 to 15 critical steps, each displayed on a separate screen consisting of an imperative title, 2 to 4 lines of text, and either a picture or video illustrating the step. In section 3, background information for critically important steps was provided in an interrogative style. At the end of each module, we offered 3 voluntary text-based multiple-choice questions as a knowledge assessment to support information retention and concept understanding.

On the opening screen of each module employees could choose between 2 options to navigate the module (i.e., browse at their own discretion or watch a narrated video showing the 3 sections in a linear fashion). Navigation within a module was by arrows at the bottom of the screen and included a “Menu” button to return to the opening screen. For a few words within some of the modules, which might not be familiar to the user, we provided a definition in a pop-up window that could be accessed by clicking on the word.

Two OSCE (i.e., treating a cow at dry-off, administering internal teat sealant) were developed according to Davis et al. (2006) and Hall et al. (2023). Both consisted of a paper-based checklist with 15 statements and 3 answer options (step performed incorrectly or correctly, does not apply). A step performed incorrectly was scored with 0, whereas a step performed correctly was scored with 1 or 2 points based on how critical the statement was to the procedure (regular vs. critical step).

A total of 20 points was possible for each OSCE. The checklists directly reflected the steps of task execution, which were described in the SOP. If possible, all employees were scored performing the procedure on 2 cows before and within 2 to 3 wk after the training was applied.

The senior author (PV) visited a convenience sample of 15 farms located in New York State to conduct the study between October and December 2021. These farms were a convenience sample based on long-term working relationships with the Quality Milk Production Services (QMPS) from the Animal Health Diagnostic Center at Cornell University and the willingness of the farm to participate in this research.

During the first visit, an extension survey was conducted to assess the milking equipment (e.g., vacuum levels, pulsators), the milker (e.g., timing, milk flow rates, unit alignment scoring), and the cows (e.g., teat scoring, strip yields, hygiene). The pre-training OSCE was performed at the time of the survey or shortly thereafter. All participants had responsibility for and experience in both procedures.

The online training was set up as a 1-h event with the employees being paid by the farm for this training. The employees were instructed to log in to the course either on their cell phone or with a tablet that we provided to complete the course during the next 50 min. A description of the terms and conditions of use and the privacy and data protection policies was provided on the first page of the course. This training project was performed following the oral consent guidance from the Cornell University Institutional Review Board for Human Participants. The farm manager or owner was also given access to the course and asked to participate. After the milkers had completed the course, we verbally asked how they liked the modules and if they had any suggestions for how to improve them. Two to three weeks after the training event, the farm was revisited on dry-off day. All employees who performed the dry-off procedure were scored using the same OSCE as in the pre-training assessment. The first name of the employee was used to match the pre- and post-training checklists but only known by the senior author and not shared with the farm management to ensure anonymity and create a safe working environment for the milkers. The OSCE scores for the employees with a pre- and post-training assessment were tested with the Shapiro-Wilk normality test and compared with a dependent samples *t*-test in SPSS for Windows (version 22.0, SPSS Inc., IBM, Ehningen, Germany).

Research has shown that many farmers believe that quality assurance is an important goal for using SOP (Bell et al., 2006). Little, however, is known about whether SOP are effective at achieving the desired outcome (Mills et al., 2020). Therefore, we set out to evaluate SOP-based online training with a pre-post assessment of milkers' skills using OSCE.

In total, 60 employees participated in the trainings with 47 and 13 of these having Spanish and English as their native language, respectively (Table 1). The majority of participants (72%) watched the video in which the text was read to them as the slides were automatically advanced. We speculate that this was due to a limited reading proficiency. In a previous study, 73% of milkers reported a preference of audio recordings in future training (Alanis et al., 2022).

**Table 1:** Results of knowledge check questions within the modules for employees of 15 farms

Module	Participants		Total number of questions		Questions correctly answered			
					Absolute		Percent	
	English	Spanish	English	Spanish	English	Spanish	English	Spanish
Collecting an aseptic milk sample	13	43	39	129	32	88	82.1	68.2
Administering intramammary treatment	11	39	33	117	29	81	87.9	69.2
Deciding on salable milk	11	39	33	117	24	78	72.7	66.7
Treating a cow at dry-off	11	35	33	105	28	60	84.8	57.1
Administering teat sealants	11	40	33	120	30	82	90.9	68.3

Although we did not require it, the majority of participants finished all 5 modules and answered the knowledge check questions at the end of each module (English-speaking employees 72.7%–90.9%, Spanish-speaking employees: 57.1%–69.9%). We interpret this as indicative of employee engagement and conclude that the 50 min time allocated for the training event was sufficient for the 5 modules. We had learned from a previous study (Alanis et al., 2022) that it is important to provide employees with dedicated time to complete the trainings even though it is online and available any time.

Data from the knowledge check question are encouraging as the majority of employees did comprehend the main points of the module and were able to correctly answer the questions. It has been shown repeatedly that online training is effective to increase knowledge (Garzon et al., 2023) or the feeling of accuracy in work performance (Hesse et al., 2019). The main scope of this study was, however, to evaluate if the employee's behavior while conducting repetitive tasks can be refined toward an established best practice by providing step-by-step job descriptions. To avoid overloading the training event with too many survey questions, which can affect completion rate, we abstained from conducting a pre- and post-training comparison of knowledge in this study. Therefore, it remains speculative if an increase in knowledge occurred. It is interesting to note, however, that there was a consistent difference in the percent answered correctly between the English versus Spanish modules. The participants selecting the Spanish modules did not do as well. This difference might be caused by fewer years of experience as milking roles tend to be filled by Spanish-speaking employees (Moore et al., 2020) while longer term, management roles tend to be filled by English speakers. Also, a lower level of literacy (Alanis et al., 2022) may have affected the knowledge assessment.

As it is human nature to deviate from protocols to make a task easier or faster, it is important to monitor employees who are doing the tasks to understand how well SOP are followed (Bauer, 2023). While several previous studies evaluated the perception or knowledge retention (Hesse et al., 2019; Garzon et al., 2023), data on compliance behavior are missing. Therefore, we used OSCE checklists to monitor employees before and after SOP-based training. Thirty-five and twenty-four employees were assessed using the treating a cow at dry-off and the administering teat sealants OSCE before completing the training, respectively. The average total OSCE score of all employees was 12.4 (±0.79) and 11.3 (±0.95) out of a possible 20 points, respectively. The scores for individuals ranged from 5 to 18 points and 6 to 18 points, respectively.

On the pre-training assessment for treating a cow at dry-off, the critical steps with the poorest implementation were in the areas of how the employees massaged the product out of the teat cistern (29% correct), putting leg bands on cows (31% correct), how they disinfected teats (34% correct), putting on new gloves (37% correct), and order of treatment (54% correct). The low percentages of correct implementation are surprising. In part, they can be explained that for this data analysis, an individual needed to have a correct score on both cows scored to be counted as having performed the statement correctly. Even though there are a plethora of studies evaluating different dry cow therapies (e.g., Vasquez et al., 2018; McCubbin et al., 2022), only a few describe the procedure in a step-by-step manner, provide evidence for the rationale of a given step, or show data on correct implementation that could be used as a reference. Furthermore, the 15 farms that participated in the study were a convenience sample where farm owners agreed to make time available for training. This type of enrollment could have introduced bias. Also, it is unclear how the presence of the senior author (PV) in the milking parlor influenced the assessment of the milkers. It is apparent that further research is warranted to elucidate these findings with a larger study enrolling more employees and farms that allows also to investigate associations with other factors.

The largest areas of opportunity identified for internal teat sealant administration involved putting leg bands on cows (21% correct), disinfecting the teats (25% correct), squeezing off the teat base during administration (25% correct), putting new gloves on (29% correct), and partial insertion of the tip of the sealant tube (38% correct). Again, we could not find any studies with which to compare these data. While there is strong evidence that internal teat sealant–based dry-off approaches are efficient for preventing new IMI during the dry period (Dufour et al., 2019), the occlusion of the teat canal proximal to the udder to maximize sealant placement within the teat canal has not been studied in controlled experiments. However, it is plausible and has been described in some studies on internal teat sealants (e.g., Bates et al., 2022). The low implementation rate illustrates how important it is to also cover simple concepts in training modules, which has also been demonstrated for calf health (Neukirchner et al., 2022) and the need to monitor procedural drift (Bauer 2023). To the best of our knowledge, no specific information is available about how training of new milkers considering critical steps is conducted and procedural compliance over time monitored.

Six employees for treating a cow at dry-off and 5 employees for administering teat sealants scored pre-training were not available on the day of the post-training assessment or had left the farm. Thus, for 29 and 19 employees, data were available for a comparison between pre- and post-training assessment OSCE scores (Table 2).

**Table 2:** Comparison of pre- and post-training scores obtained with objective structured clinical examinations (OSCE) from 15 farms

OSCE score	Treating a cow at dry-off	Administering internal teat sealant
Participants (n)	29	19
Pre-training OSCE score (mean ± SE)	12.4 ± 0.79^a^	11.3 ± 0.95^a^
Post-training OSCE score (mean ± SE)	15.3 ± 0.49^b^	13.7 ± 0.95^b^
Mean difference score (mean ± SE)	3.0 ± 0.49	2.4 ± 0.51
Participants with scores worsened (%)	3	5
Participants with scores unchanged (%)	14	5
Participants with scores improved (%)	83	90

a,b Values within the same column with different superscripts differ (*P* < 0.001).

There was an improvement in the average (±SE) OSCE score for both modules (i.e., treating a cow at dry-off, administering an internal teat sealant) from 12.4 (±0.79) and 11.3 (±0.95) pre-training to 15.3 (±0.49) and 13.7 (±0.93) post-training (*P* < 0.001) out of a total of 20 possible points. The mean differences between pre- and post-training scores were 3.0 ± 0.49 and 2.4 ± 0.51. Considering individual participants, there was an improvement after the training for 83% and 90% of the participants. The second assessment was conducted 2 to 3 wk after the training event, which is a relatively short interval considering the observation that training takes place only once or twice a year (Durst et al., 2018; Neukirchner et al., 2022). It would be interesting to investigate when procedural drift actually occurs and when retraining is indicated.

Effective training programs that provide dairy employees with the necessary knowledge and skills to make health and production-related decisions are absolutely critical (Roman-Muniz et al., 2007). Interestingly, it has been shown that owners or managers underestimate employees' interest in regular training to improve skills (Durst et al., 2018). To our knowledge this is the first study using OSCE to assess milker behavior for relevant tasks and to demonstrate that behavior can be changed with SOP-based online courses toward a desired manner. Further research is needed to study factors that might enhance or hinder training success, knowledge retention, and behavioral change. Also, we want to study if a reduction of items on the checklist is possible without compromising accuracy to simplify the assessments.
